# Intravascular Ultrasound-Guided Versus Angiography-Guided Percutaneous Coronary Intervention: A Systematic Review and Meta-Analysis

**DOI:** 10.7759/cureus.69167

**Published:** 2024-09-11

**Authors:** Gautham Varun Krishna Mohan, Nawabzada Nadir Babar, Sindhuja Sompalli, Muhammad Umar Mian, Farhan Israr, Sandipkumar S Chaudhari, Calvin R Wei, Danish Allahwala

**Affiliations:** 1 Internal Medicine, Tirunelveli Medical College, Tirunelveli, IND; 2 Internal Medicine, Combined Military Hospital (CMH) Lahore Medical College and Institute of Dentistry, Lahore, PAK; 3 Internal Medicine, JSS Medical College, Hyderabad, IND; 4 Internal Medicine, Allama Iqbal Medical College, Lahore, PAK; 5 Medicine, Khyber Medical College, Peshawar, PAK; 6 Internal Medicine, Lady Reading Hospital, Peshawar, PAK; 7 Cardiothoracic Surgery, University of Alabama at Birmingham, Birmingham, USA; 8 Family Medicine, University of North Dakota School of Medicine and Health Sciences, Fargo, USA; 9 Research and Development, Shing Huei Group, Taipei, TWN; 10 Nephrology, Fatima Memorial Hospital, Karachi, PAK

**Keywords:** angiography-guided, angiography-guided pci, cardiovascular outcomes, intravascular imaging-guided, intravascular imaging-guided pci, percutaneous coronary intervention, systematic review and meta analysis

## Abstract

This meta-analysis evaluated the clinical outcomes of intravascular ultrasound (IVUS)-guided versus angiography-guided percutaneous coronary intervention (PCI) in patients with coronary artery disease (CAD). A comprehensive literature search was conducted across major electronic databases, identifying relevant studies published up to August 15, 2024. Thirteen randomized controlled trials (RCTs) met the inclusion criteria, comparing IVUS-guided and angiography-guided PCI. The primary outcomes were major adverse cardiac events (MACE) and stent thrombosis, while secondary outcomes included all-cause mortality, cardiac mortality, myocardial infarction, and revascularization rates. Pooled analysis revealed that IVUS-guided PCI significantly reduced the risk of MACE (risk ratio (RR): 0.63, 95% CI: 0.50-0.79) and stent thrombosis (RR: 0.52, 95% CI: 0.30-0.90) compared to angiography-guided PCI. Secondary outcomes also favored IVUS guidance, with significant reductions in cardiac mortality, myocardial infarction, target lesion revascularization (TLR), and target vessel revascularization (TVR). While a trend towards reduced all-cause mortality was observed with IVUS guidance, it did not reach statistical significance. Notably, low heterogeneity across studies strengthened the reliability of these findings. Meta-regression analysis indicated that the presence of myocardial infarction did not significantly moderate the effect of IVUS on clinical outcomes, suggesting consistent benefits across patient subgroups. These results highlight the potential of IVUS-guided PCI to improve cardiovascular outcomes and reduce the need for repeat procedures. The findings support the growing body of evidence favoring IVUS use in PCI, particularly in complex lesions and high-risk patients. However, considerations such as cost-effectiveness and the need for specialized training remain important factors in the widespread adoption of IVUS-guided PCI in clinical practice.

## Introduction and background

Percutaneous coronary intervention (PCI) is a cornerstone in the management of coronary artery disease (CAD), aimed at alleviating ischemia and improving patient outcomes by restoring adequate blood flow through stenotic coronary arteries [1]. Traditionally, PCI has been guided by angiography, a widely accessible imaging technique that provides two-dimensional visualization of the coronary lumen [2]. However, angiography has notable limitations, including its inability to accurately assess the extent of plaque burden, the characteristics of the arterial wall, and the precise dimensions of the vessel [3]. These limitations can lead to suboptimal stent placement, incomplete lesion coverage, or even inadvertent injury to the vessel, which can contribute to adverse clinical outcomes such as restenosis, stent thrombosis, and recurrent ischemic events [4]. 

Intravascular ultrasound (IVUS) offers an alternative imaging modality that has the potential to overcome many of the shortcomings associated with angiography-guided PCI [5]. An IVUS provides high-resolution, cross-sectional images of the coronary arteries, enabling detailed visualization of both the lumen and the vessel wall. This advanced imaging capability allows for a more precise assessment of plaque morphology, accurate measurement of vessel dimensions, and optimal stent selection and deployment [6]. Consequently, IVUS-guided PCI has been proposed to reduce the risk of procedural complications and improve long-term clinical outcomes [5]. 

Despite the theoretical advantages of IVUS, its routine use in PCI remains a subject of ongoing debate. The additional costs associated with IVUS, extended procedure times, and the need for specialized expertise are often cited as barriers to its widespread adoption [7]. Moreover, while several studies have suggested that IVUS guidance may lead to better outcomes compared to angiography-guided PCI, the evidence remains mixed, with some studies showing no significant difference in key clinical endpoints [8]. This uncertainty underscores the need for a comprehensive evaluation of the available evidence to determine the true impact of IVUS on PCI outcomes. 

The present meta-analysis seeks to systematically review and synthesize data from studies comparing IVUS-guided and angiography-guided PCI. By pooling results from a diverse range of clinical trials, this analysis aims to provide a robust comparison of the efficacy, safety, and long-term outcomes associated with each approach. Specifically, the meta-analysis will focus on key clinical endpoints, including rates of major adverse cardiac events (MACE), stent thrombosis, and all-cause mortality. The findings of this study are expected to contribute to the ongoing debate regarding the optimal guidance strategy for PCI and may have important implications for clinical practice, particularly in determining the role of IVUS in enhancing the precision and effectiveness of PCI procedures. 

## Review

Methodology 

Search Strategy 

To ensure a thorough and unbiased selection of studies for our meta-analysis, we implemented a comprehensive literature search strategy. Our team conducted systematic searches across multiple prominent electronic databases, including but not limited to PubMed, the Excerpta Medica database (EMBASE), Web of Science, and the Cochrane Library. We focused on articles published through August 15, 2024, without imposing restrictions on language or publication status. Our search methodology incorporated a diverse array of relevant terms, encompassing both keywords and Medical Subject Headings (MeSH). These included variations of "Intravascular Ultrasound," "Angiography," "Percutaneous Coronary Intervention," "Stent," and "Coronary Artery Disease," among others. To optimize search efficiency and precision, we employed Boolean operators to construct complex queries. To maximize coverage, we supplemented our electronic database searches with a manual review of reference lists from included studies and pertinent review articles. This approach allowed us to capture potentially relevant studies that may have eluded our initial database searches. The search process was independently executed by two members of our research team. In cases of disagreement regarding study inclusion, resolution was achieved through collaborative discussion and, when necessary, consultation with senior investigators. This dual-reviewer approach helped mitigate potential selection bias and ensure the robustness of our study identification process. 

Study Selection 

The selection process followed predefined inclusion and exclusion criteria. Studies were included if they were randomized controlled trials (RCTs) that directly compared IVUS-guided PCI with angiography-guided PCI in patients with coronary artery disease. Only studies that reported on clinical outcomes such as MACE, stent thrombosis, or mortality, cardiovascular mortality, myocardial infarction, target lesion revascularization (TLR), and target vessel revascularization (TVR) were considered. Exclusion criteria included studies that lacked a comparative design, those that did not report relevant outcomes and duplicate publications. Two independent reviewers screened the titles and abstracts of all identified articles, followed by full-text reviews of potentially eligible studies. Discrepancies between reviewers were resolved through discussion, and a third reviewer was consulted when necessary. 

Data Extraction and Outcomes 

To extract data systematically and minimize bias, we employed a rigorous dual-reviewer approach. A customized data extraction form was developed to capture essential information from each study, including methodological details (such as author names, publication dates, study designs, participant numbers, and follow-up periods), participant profiles (focusing on age, gender distribution, and myocardial infarction presentation), and reported clinical outcomes. Our primary interest lay in MACE and stent thrombosis, while secondary outcomes encompassed overall and cardiovascular-specific mortality, myocardial infarction recurrence, and revascularization rates for both target lesions and vessels. The extraction process involved one team member initially populating the form, followed by a thorough cross-verification by a second reviewer. This meticulous approach ensured data accuracy and completeness, with any inconsistencies promptly addressed through collaborative discussion among the research team. 

*Data Analysis* 

Our statistical analysis was conducted using a combination of software tools: RevMan 4.5.1 (The Cochrane Collaboration, London, UK) and STATA 17.0 (StataCorp LLC, College Station, TX). To compare outcomes between the two study groups, we calculated risk ratios (RRs) with their corresponding 95% CIs. Statistical significance was established at a p-value threshold of 0.05. To assess the consistency of results across studies, we employed the I-squared statistic as a measure of heterogeneity. When I-squared values exceeded 50%, indicating substantial heterogeneity, we opted for a random-effects model in our pooled analysis. Conversely, in cases of low heterogeneity (I-squared < 50%), we utilized a fixed effects model for data synthesis. To further explore the data, we conducted meta-regression analyses. These analyses aimed to investigate whether the presence of myocardial infarction moderated the relationship between IVUS utilization and the observed clinical outcomes. 

Results

**Figure 1:**
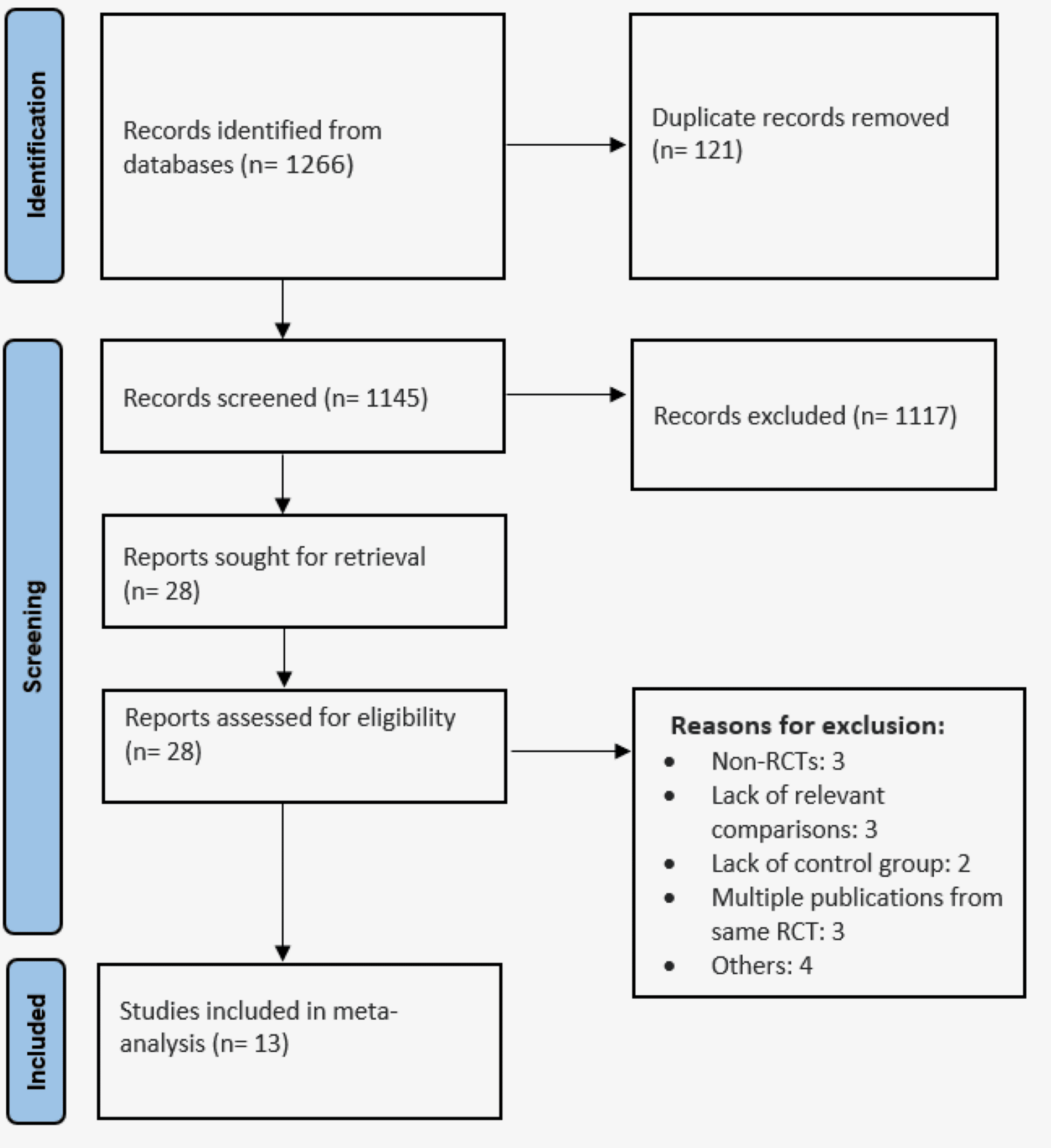
A PRISMA flowchart representing process of study selection PRISMA: Preferred Reporting Items for Systematic Reviews and Meta-Analyses; RCT: randomized controlled trial

**Figure 2:**
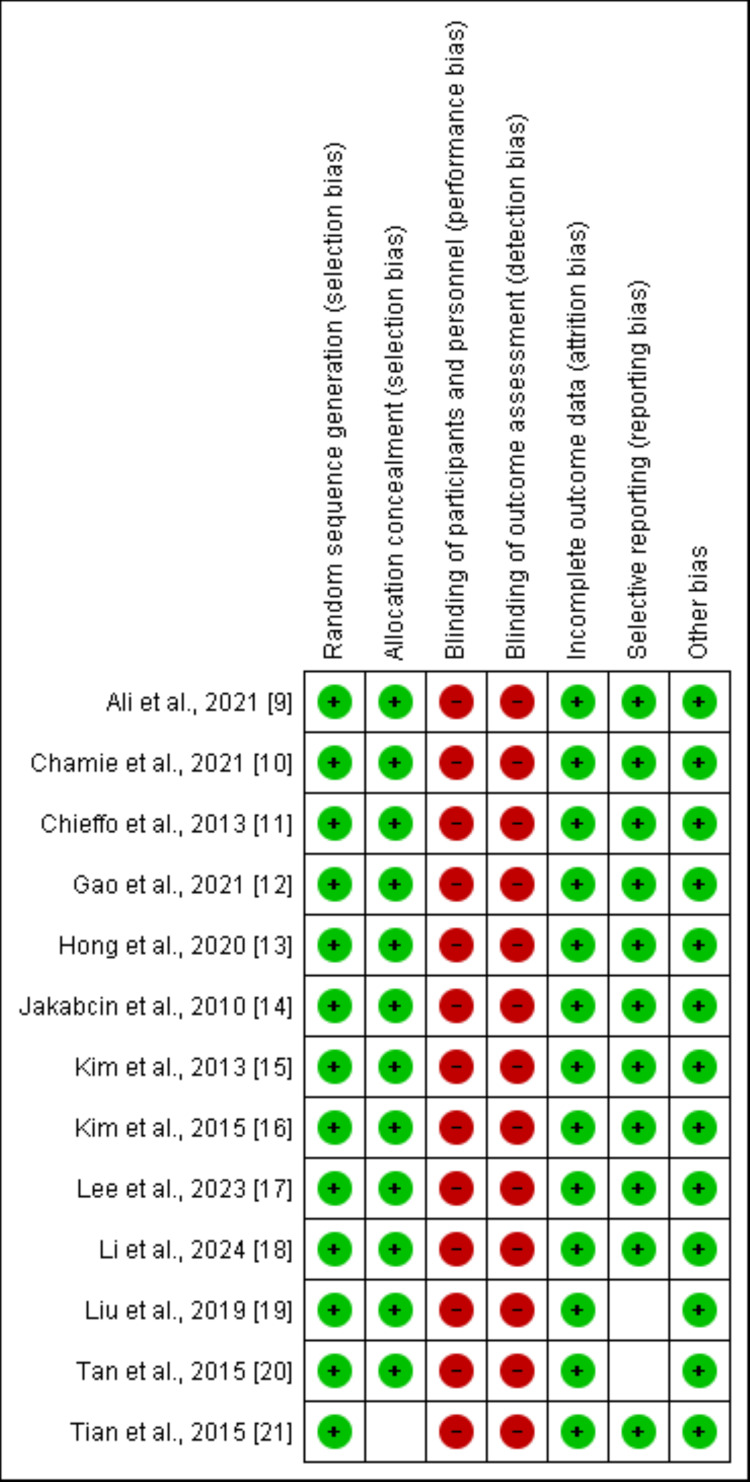
Risk of bias assessment of the included studies

Online database searching yielded 1,266 records. We removed duplicates and initially screened 1,145 records based on inclusion and exclusion criteria. We removed 1,117 studies as they were not relevant to the study objective (most papers were either editorials or review papers). A detailed assessment of 28 studies was done after getting the full text of these articles. Based on eligibility criteria, 13 studies were included in this meta-analysis. Figure 1 shows the study selection process. Table 1 shows the characteristics of the included studies. Follow-up of included studies ranged from 12 months to 60 months. The included studies were published from 2010 to 2024. Figure 2 shows the risk of bias assessment of included studies performed using the Cochrane Risk of Bias (RoB) assessment tool.

**Table 1:** Study and participants characteristics RCT: randomized controlled trial; MI: myocardial infarction; NR: not reported

Author	Year	Design	Groups	Sample size	Follow-up	Age (years)	Males (n)	MI (n)
Ali et al. [9]	2021	RCT	Intravascular ultrasound-guided	136	12 months	NR	NR	NR
			Angiography-guided	142				
Chamie et al. [10]	2021	RCT	Intravascular ultrasound-guided	50	30 months	59.32	36	10
			Angiography-guided	49		58.59	38	12
Chieffo et al. [11]	2013	RCT	Intravascular ultrasound-guided	142	24 months	63.9	117	NR
			Angiography-guided	142		63.6	109	
Gao et al. [12]	2021	RCT	Intravascular ultrasound-guided	724	36 months	65.2	535	NR
			Angiography-guided	724		65.9	530	
Hong et al. [13]	2020	RCT	Intravascular ultrasound-guided	589	60 months	63	408	87
			Angiography-guided	594		63	409	98
Jakabcin et al. [14]	2010	RCT	Intravascular ultrasound-guided	105	18 months	59.4	73	60
			Angiography-guided	105		60.2	71	72
Kim et a.l [15]	2013	RCT	Intravascular ultrasound-guided	269	12 months	62.8	177	24
			Angiography-guided	274		64.3	150	27
Kim et al. [16]	2015	RCT	Intravascular ultrasound-guided	201	12 months	61	162	NR
			Angiography-guided	201		61.4	162	
Lee et al. [17]	2023	RCT	Intravascular ultrasound-guided	1092	25.2 months	65.3	869	199
			Angiography-guided	547		66	431	99
Li et al. [18]	2024	RCT	Intravascular ultrasound-guided	1753	12 months	62	1285	1054
			Angiography-guided	1752		63	1299	1026
Liu et al. [19]	2019	RCT	Intravascular ultrasound-guided	167	12 months	65.3	106	17
			Angiography-guided	169		64.9	108	21
Tan et al. [20]	2015	RCT	Intravascular ultrasound-guided	61	24 months	76.54	38	0
			Angiography-guided	62		75.85	43	0
Tian et al. [21]	2015	RCT	Intravascular ultrasound-guided	115	24 months	NR	NR	NR
			Angiography-guided	115				

Major Adverse Cardiac Events

**Figure 3:**
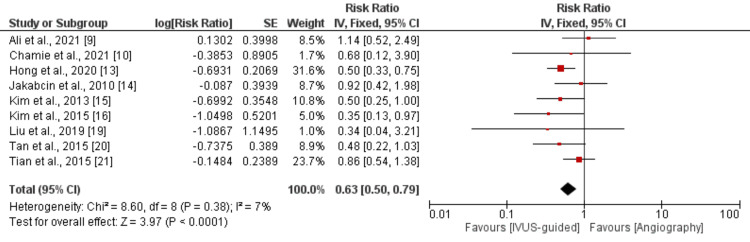
Comparison of all-cause death following intravascular imaging–guided PCI and angiography-guided PCI PCI: percutaneous coronary intervention References: [9-10, 13-16, 19-21]

We analyzed MACE by conducting a pooled analysis of nine studies, and the results of the pooled analysis are shown in Figure 3. Pooled analysis showed that the risk of MACE was significantly lower in the IVUS-guided group compared to patients randomized in the angiography-guided group (RR: 0.63, 95% CI: 0.50-0.79, p-value<0.001). Low heterogeneity was reported among the study results (I-square: 7%).

Stent Thrombosis 

**Figure 4:**
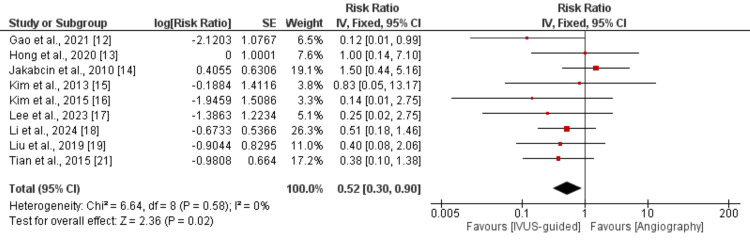
Comparison of stent thrombosis following intravascular imaging-guided PCI and angiography-guided PCI PCI: percutaneous coronary intervention References: [12-19, 21]

We analyzed stent thrombosis by conducting a pooled analysis of nine studies, and the results of the pooled analysis are shown in Figure 4. Pooled analysis showed that the risk of developing stent thrombosis was significantly lower in the IVUS-guided group compared to patients randomized in the angiography-guided group (RR: 0.52, 95% CI: 0.30-0.90, p-value<0.001). Low heterogeneity was reported among the study results (I-square: 0%). 

Secondary Outcomes 

Table 2 presents a comparison of secondary outcomes between the two groups. These results suggest that IVUS-guided PCI may offer significant clinical benefits compared to angiography-guided PCI across several important outcomes. While there was no statistically significant difference in all-cause death (RR 0.81, 95% CI 0.61-1.07), IVUS guidance was associated with a 50% reduction in cardiac mortality (RR 0.5, 95% CI 0.36-0.71). Furthermore, IVUS use was linked to lower rates of myocardial infarction (26% reduction), target lesion revascularization (39% reduction), and target vessel revascularization (42% reduction). Notably, the absence of heterogeneity (I-squared 0% for all outcomes) suggests consistent findings across studies, strengthening the reliability of these results. These data indicate that IVUS guidance during PCI may lead to improved cardiovascular outcomes and reduced need for repeat procedures. 

**Table 2:** Comparison of secondary outcomes following intravascular imaging–guided PCI and angiography‐guided PCI TLR: target lesion revascularization; TVR: target vessel revascularization: CI: confidence interval; PCI: percutaneous coronary intervention * Significant at a p-value less than 0.05

Outcomes	Number of studies	Risk ratio	95% CI	I-square
All-cause death	8	0.81	0.61 to 1.07	0%
Cardiac mortality	11	0.5	0.36 to 0.71*	0%
Myocardial infarction	12	0.74	0.60 to 0.92*	0%
TLR	11	0.61	0.49 to 0.75*	0%
TVR	9	0.58	0.47 to 0.72*	0%

Meta-regression 

Meta-regression analysis was conducted to evaluate the potential moderating effect of myocardial infarction on the relationship between IVUS use and various clinical outcomes. The outcomes examined included MACE, stent thrombosis, all-cause mortality, cardiac mortality, myocardial infarction, TLR, and TVR. The results of the meta-regression, as presented in Table 3, indicate that the presence or proportion of patients with myocardial infarction in the studies did not significantly influence the effect of IVUS on any of the examined outcomes. This suggests that the benefits or effects of IVUS-guided PCI appear to be consistent across different levels of myocardial infarction prevalence in the study populations. These findings imply that the efficacy of IVUS in improving clinical outcomes is not substantially modified by the presence of myocardial infarction. Therefore, the potential benefits of IVUS-guided PCI observed in the primary analysis may be applicable across a broad spectrum of patients, including those with and without myocardial infarction.

**Table 3:** Meta-regression to assess the impact of myocardial infarction on outcomes MACE: major adverse cardiovascular events; TLR: target lesion revascularization; TVR: target vessel revascularization

Outcomes	Coefficient	P-value
MACE	0.00026	0.894
Stent thrombosis	-0.00024	0.49
Death	-0.0001	0.652
Cardiac mortality	-0.0002	0.983
Myocardial Infarction	-0.0004	0.525
TLR	0.00022	0.443
TVR	0.00015	0.685

Discussion

This meta-analysis was conducted to compare the effectiveness of IVUS-guided PCI with angiography-guided PCI. A total of 13 studies were included in the analysis. The results demonstrated that primary outcomes, specifically MACE and stent thrombosis, were significantly lower in patients who underwent IVUS-guided PCI. Furthermore, secondary outcomes, including all-cause mortality, cardiovascular mortality, TLR, TVR, and myocardial infarction, were also less likely to occur in patients receiving IVUS-guided PCI compared to those who underwent angiography-guided PCI. The effect of IVUS-guided PCI is large, and we observed low or no heterogeneity for all the outcomes, including in this meta-analysis that showed a consistent effect across all the included studies. A meta-analysis conducted by Sreenivasan et al. reported a similar advantage of IVUS-guided PCI over angiography-guided PCI [22]. 

Improved clinical outcomes with intravascular imaging-guided PCI, such as IVUS, are largely due to the precise implantation of larger stents with greater final minimal stent areas [23]. Unlike angiography-guided PCI, which provides only a two-dimensional view of the vessel, IVUS offers detailed cross-sectional imaging, enabling accurate stent sizing and ensuring optimal expansion, thereby reducing the risk of restenosis [24]. Additionally, IVUS helps avoid leaving significant plaque burden at the stent edges and identifies untreated edge dissections, which are often missed with angiography [25]. By allowing for better visualization and more precise interventions, IVUS-guided PCI significantly enhances the safety and effectiveness of stent placement compared to angiography-guided PCI [26]. 

The guidelines of IVUS and angiography-guided PCI have evolved with emerging evidence. While angiography remains the standard for routine cases, major cardiology societies, including the American College of Cardiology (ACC)/American Heart Association (AHA), European Society of Cardiology (ESC), and Society for Cardiovascular Angiography and Interventions (SCAI), recommend IVUS for specific scenarios. These include assessing left main coronary artery disease, evaluating intermediate stenoses, and optimizing stent deployment in complex lesions [27]. An IVUS is particularly useful for bifurcations, long lesions, and small vessels. Guidelines generally support IVUS use in selected cases but don't mandate its routine application in all PCIs, considering factors like cost and additional training requirements [28]. Recommendations are typically Class IIa or IIb, indicating that IVUS is reasonable or may be considered in certain situations, with the final decision often left to the operator's discretion [27]. 

Our findings indicate a potential advantage in overall mortality, though this outcome did not reach statistical significance. The point estimate for all-cause death favored the use of intravascular imaging compared to angiography across the entire patient population. We posit that an intervention demonstrating significant reductions in major adverse cardiac events, cardiac mortality, stent thrombosis, and various revascularization measures to the degree shown by intravascular imaging is likely to contribute to a decrease in overall mortality. However, our current analysis lacks sufficient statistical power to definitively prove this benefit. As additional studies are conducted and more patient data becomes available, we anticipate that the precision of our estimates will improve, potentially leading to statistically significant results regarding the reduction in all-cause mortality. 

We performed meta-regression to understand the potential moderating effect of myocardial infarction on the relationship between IVUS use and various clinical outcomes. We did not find any significant impact of myocardial infarction. Some studies have shown that IVUS-guided PCI can be particularly beneficial in patients with acute myocardial infarction (AMI). For instance, a meta-analysis by Lin et al. found that IVUS-guided PCI was associated with lower rates of MACE and cardiac death in AMI patients compared to angiography-guided PCI [29]. 

Study limitations 

This meta-analysis has certain limitations. Firstly, we were not able to perform subgroup analysis due to lack of data. However, we have performed meta-regression to understand the potential moderating effect of myocardial infarction on the relationship between IVUS use and various clinical outcomes. Secondly, the inconsistency in definitions of clinical outcomes and subgroups across the included trials is a common issue in all meta-analyses, presenting a challenge to achieving uniformity in analysis. 

## Conclusions

Based on the comprehensive meta-analysis presented, IVUS-guided PCI demonstrates significant clinical advantages over angiography-guided PCI. The findings show reduced rates of major adverse cardiac events, stent thrombosis, cardiac mortality, myocardial infarction, and revascularization procedures with IVUS guidance. The consistency of these results across studies, as indicated by low heterogeneity, strengthens the reliability of these findings. While all-cause mortality showed a trend favoring IVUS, it did not reach statistical significance, possibly due to limited statistical power. The benefits of IVUS appear to be consistent regardless of myocardial infarction status. These results suggest that wider adoption of IVUS-guided PCI could potentially improve outcomes in coronary interventions, though considerations of cost and training requirements remain important factors in its implementation.
